# Inclusive and collaborative advanced transport: are we really heading to sustainable mobility?

**DOI:** 10.1186/s12544-022-00570-1

**Published:** 2022-10-21

**Authors:** Pierluigi Coppola, António Lobo

**Affiliations:** 1 Department of Mechanical Engineering, Politecnico di Milano, Via G. La Masa 1, 20156 Milan, Italy; 2 CITTA – Research Centre for Territory, Transports and Environment, Faculty of Engineering, University of Porto, Rua Dr. Roberto Frias, s/n, 4200-465 Porto, Portugal

## Introduction

This Topical Collection of European Transport Research Review includes a selection of papers presented at the 48th European Transport Conference (ETC), organized on-line by the Association for European Transport (AET), from September 7th to September 9th 2020. The ETC is a major annual event where European transport practitioners and researchers come together to keep abreast of policy issues, research findings and best practices across a broad spectrum of transport topics: from advanced modelling for passenger and freight transport to appraisal methods; from sustainable planning to public transport and rail case studies. Uniquely in Europe, the Conference provides a forum for those engaged in research, policy and business in transport, bridging the gap that often arises between theory and practice.

This Topical Collection on Inclusive and Collaborative Transport brings together a number of contributions to reflect on how to promote an effective and sustainable transition toward new forms of mobility, including advanced level of automation and digitalization. In fact, thanks to the digital transformation, new transport modes and mobility services are now available (e.g., ride‑hailing, car sharing, demand-responsive transport systems, and micro-mobility) or will be available in the future (e.g., fully automated and connected vehicles), paving the way to a wider range of options to improve accessibility to places nowadays accessible only by private transport. Moreover, this creates the opportunities to supply safer and more suitable transport services also for users with limited access to transport (e.g., elderly, young, umpired and other vulnerable users).

However, as the complexity of the transport system increases on both the demand and the supply side, new challenges arise for transport researchers, planners, and developers. For each innovative solution that succeeds, many concepts fail at different development stages or even after deployment, because they were not technically feasible, economically viable, environmentally sustainable, or simply because they were ahead or behind of their time [14, 42]. Moreover, the development and the implementation on a large scale of new transport solutions ought to confront also with their social acceptance, that in turn requires to understand needs, fears, and behaviours of different communities and groups of population [10, 11]. To this aim, community-led initiatives and public participation [22, 27] could help technicians and policymakers to ensure a smooth (and accepted) integration of new breakthroughs in the transport ecosystem. However, a collaborative environment does not always prioritize the common good, as the main driver of innovation has naturally commercial goals beyond social responsibility.

This Topical Collection includes 11 papers that can advance the research on collaborative approaches and inclusive solutions for transport systems, exploring new fields and proposing up-to-date policy recommendations to govern the transition towards future sustainable mobility scenarios from a strongly car-dependent society.

## Old habits and a new reality

According to the Eurostat [25], the modal split of passenger transport in Europe is largely dominated by cars, which account for nearly 83% of the passenger-km measured in the European Union (EU). The remaining share is almost evenly distributed by rail (8%) and bus (9%). There are some differences between countries, as, for instance, in 2019, Hungary was the least car dependent country (72%), while Lithuania was the most (91%), but the EU-average has remained stable at least for the last two decades. When considering the mode choice for the most frequent trip, in many cases commuting trips, the results of a European-wide survey carried out in 2014 [13] showed that the car represented less than 50% of those trips only in nine countries out of the 28 EU countries at the time. Other important conclusions from this survey reflecting a strong European car culture are that 82% of the respondents hold a driving license, and that the EU-average car ownership is above 0.5 registered cars per person, even in metropolitan areas and among low-income respondents (peaks at approximately 0.75 in rural areas and 0.9 among high-income citizens). Over the last 20 years, car ownership soared in Eastern European countries belonging to the EU, where an average increase of 69% was observed in comparison with 31% observed in the countries that accessed the EU before this millennium [28].

These figures show that the comfort and flexibility of private cars still make them very attractive for those who can afford ownership costs [13], despite the numerous policies and measures to restrict the use of cars and to promote public transport and active modes. Beirão and Cabral [2], who made a very comprehensive review of the reasons behind travellers choosing the car over more sustainable modes, advocate that identifying the groups of car users that are most motivated to change and defining policies specially targeted at those groups can be much more effective than investing in the overall expansion/improvement of the alternative modes.

Taking the example of shopping, two papers included in this TC established a direct connection between their findings and car dependence. Rokseth et al. [31] noted that private cars have for a long time been the main mode of transport for retail and service trips, of which 60% correspond to grocery shopping. Therefore, the authors conducted a spatial analysis based on Geographic Information Systems (GIS) in two Norwegian towns to conclude that the percentage of population living close to a grocery shop (i.e., at a walking distance of less than 500 m) has nearly halved over the last 40 years. The gradual replacement of small neighbourhood shops by large retail stores, typically located in the suburbs, together with a large increase of the country’s motorization rate, have further promoted the car use for basic daily activities such as grocery shopping. In the end, the authors question whether spatial planning is actually contributing to a car-free future.

Bönisch et al. [4] also acknowledge the importance of cars in shopping trips, but hypothesize whether new e-commerce services can be one more argument to adopt a car-free lifestyle. In this study, a latent class analysis was performed with a sample of 466 residents in Munich, Germany, who were interviewed about their online and in-store shopping and travel behaviour. Results show that those who feel more attracted by online shopping also use their cars frequently in their daily routines, including for traveling longer distances for grocery shopping. The use of the car for going shopping still seems to depend more on the individual’s socioeconomic characteristics, place of residence, and shopping motivation/daily needs than on the availability of more online shopping platforms.

Although this Topical Collection does not include any paper that addresses the impacts of the COVID-19 pandemic, as the research on this topic was still at its early stages at the time of the submissions to the 48th ETC, it is now becoming evident that such disruptive event changed the way we live, work and commute, with effects that may last for a long time [38]. When the COVID-19 disease, caused by the new coronavirus SARS-Cov-2, was officially declared a pandemic by the World Health Organization in March 2020 [43], many countries and cities around the world imposed strict lockdowns to contain the virus spread. These massive lockdowns, telecommuting, home quarantines, and voluntary self-isolation had naturally a strong impact on transport and mobility, with all transport modes suffering from a sharp decline in ridership [9, 23]. As the prevention and treatment of COVID-19 has seen remarkable developments since the beginning of the pandemic [3, 34] and the now dominant Omicron variant seems less severe [16], many health protective measures and travel restrictions have been lifted or, at least, alleviated in most countries around the world. As a result, travel demand has registered an unprecedented overall contraction, dramatically dropping down with peaks of less 90 to 95% passengers for public transport. Nowadays, demand by car has resumed the levels before the crisis, although some structural changes have been observed in public transport. The risks of contagion are still affecting the perception of transport supply and the long-term mobility impacts of the pandemic are still uncertain [38]. On the one hand, many new technology and policy instruments created during the pandemic to facilitate remote activities (e.g., working, studying, and shopping) are expected to persist in the future [5, 21], reducing the need to travel. On the other hand, the United Nations recently declared that the pandemic is far from over and that virus mutations are of great concern and may pose severe health risks [16, 41]. As some authors pointed out, socioeconomic factors play an important role in self-protecting behaviour towards the virus [26, 40], so it is likely that those who can afford it continue to avoid public transport and choose the car, counteracting the objectives of social inclusion. In other words, it is still unclear whether this new reality can strengthen the old car-dependency habits and postpone the transition to more sustainable forms of transportation.

## Infrastructure and accessibility gaps

The lack of infrastructure (and, particularly, of mass rapid transit ones) has always been identified as one of the major causes of car dependency, and therefore of social exclusion and inequitable access to opportunities (e.g., jobs, healthcare, education). However, also in areas with good endowment of metro and tram lines, it has been observed a persistent predominance of car use due to the increasing complexity of travellers’ trip chains that requires flexible transport services and seamless door-to-door travel solutions for multiple origin and destination journeys.

In this Topical Collection the accessibility problems have been studied at very different scales of analysis. Mocanu et al. [24] developed a country-wide study in Germany to investigate the potential for a mode shift from car to public transport in commuting trips, based on travel-time accessibility measures. The potential for a mode shift appears to be very low, since the average travel time by public transport is almost three times more than by car. This conclusion is largely independent of the type of region (metropolitan, medium- and small-size cities, or rural). Guida et al. [15] analysed spatial accessibility to healthcare services at the metropolitan scale in Milan, Italy, focusing on residents with 65 years old or more, i.e., those who are more likely to need healthcare assistance. Using a GIS environment, the authors computed a multi-component accessibility measure for three age subgroups (65–69, 70–74, and 75 + years old) based on land use, mobility features, and elderlies’ behavioural traits. It was possible to observe that in suburban/peripheral areas, the accessibility to healthcare services is good for a higher percentage of the elderly population in relation to semi-central and central areas. Although there are less services in the periphery, a higher percentage of the population lives close to them, probably because those services are located in small villages where the population is concentrated. Surprisingly, a much larger number of people living in the central areas have poor accessibility to healthcare services by walking, bus or metro, making them more dependent on cars. In turn, Swift et al. [39] focused on analysing the value of inclusive design in the access to railway stations in the UK. A quantitative analysis showed evidence of a positive correlation between the number of persons with reduced mobility using a given train station and the station’s accessibility score, derived from the existence of step-free access to platforms and trains. A qualitative analysis based on the consultation of policymakers, private sector organisations, and transport disability advocacy groups highlighted the wider positive impacts of easy access on the attractiveness of the rail network that extend beyond the individual benefits for the passengers with reduced mobility.

The analysis of travel behaviour can also be useful to infer accessibility gaps. In this sense, Ek et al. [12] conducted a survey targeting the commuting patterns using active modes, the socioeconomic characteristics, and the health and environmental concerns of people living in metropolitan, mid-size, or small agglomerations in Sweden. A binary logit model was used to analyse the motives for walking and cycling. The results indicate that the choice of active modes is related to the commuting distance, availability of adequate infrastructure, and health and environmental concerns. Active modes seem to be a more popular commuting choice in mid-sized cities, where sidewalks and cycle lanes are better evaluated by the users than in metropolitan and rural areas. In metropolitan areas, a better public transport and a higher income may shift preferences towards motorized transport, while in rural areas the limited access to public transport and the higher attractiveness of car use that seem to discourage the use of active modes. Lemonde et al. [18] conducted a spatiotemporal analysis of multimodality indices against available context to detect zones within the city of Lisbon lacking adequate supply in specific time periods and featuring imbalanced preferences towards specific transport modes. The research was based on ridership data from bus, metro, and bike-sharing systems, and the indices were developed to assess multimodality vulnerabilities both at the passenger and the spatial levels. Generally, the authors observed two major sources of multimodal penalties: a strong preference towards the metro in the city centre, and the presence of many peripheral zones served only by bus. Also using Lisbon as case study and similar data sources, Cerqueira et al. [6] proposed a methodology to integrate situational context in the descriptive and predictive models of traffic data. The results showed that the quantifiable impacts of large public events, adverse weather, or traffic interdictions can be used to produce correction factors for a context-sensitive modelling of origin–destination matrices, traffic demand series, or raw individual trip records along the public transport system. In this way, this study aims to contribute to the mitigation of short-term impacts of sudden or planned disruptions, as well as to learn from the impacts of such disruptions towards the improvement of the accessibility to key venues/infrastructures and the resilience of the transport network.

It has been observed [8] that infrastructural interventions on public transport, although improving accessibility and reducing the generalized cost of traveling, allow only for slight reductions of private vehicles travelled distances, whereas, a profound change in the modal split may occur with the implementation of very strict travel demand management policies, such as the implementation of car-free areas. These measures coupled with the deployment of new modes of transport (e.g., shared vehicles) and “feeder” services to station and terminals, enabled by widespread diffusion of Information and Communications Technologies (ICT), can compensate the reduction of accessibility by car and may encourage a more intensive use of collective transport modes, even in areas with low demand density. In other words, the innovative and shared-mobility solutions may induce a significant modal diversion from private vehicles if properly designed to improve the access to public transport services.

## ICT and new forms of transportation

Undeniably, digital services and technology innovations have the potential to attract new users and make life easier for those who, for some economic, environmental or physical reason, face some sort of transport-related exclusion [17]. However, such a fast technology progress also raises fears of increased digital illiteracy and greater inequality between the high and the low ends of social stratification [29], as the emerging transport modes and services may be too expensive for the most unprivileged groups [1]. That is why the next big leap for technology innovation, already called the Fifth Industrial Revolution, demands for a human-centric approach in which personalization, purpose, ethics and inclusivity are key [20]. In transport research, human factors are currently more important than ever, as it is crucial to understand the people’s needs and mental representations of new technologies, as well as to prevent the risks of misuse and disuse [32].

In this Topical Collection, there are three papers that approach the preferences of the users and the new risks introduced by emerging forms of transportation. Stam et al. [36] analysed the preferences of travellers for existing and new first/last mile transport modes. To do that, a survey was conducted among the users of the Almere Centrum train station, the main public transport interface in the city of Almere, Netherlands. Four scenarios were designed to combine options for individual travel (either private or shared vehicles) with options for ride sharing (either traditional/scheduled or on-demand services). Even though walking remains a top preference, especially for activity-end trips, the preference for car-based modes increases drastically with the offer of shared vehicles and individual on-demand riding services. A large share of those potential users is poached from the bus. Younger people, women and those travelling for study/school purposes are more prone to prefer the car, which is also a popular future choice among those who do not currently own a car or do not have driver’s license. Curiously, older people are more receptive to innovative on-demand ride services that facilitate their accessibility.

In turn, the research of Soares et al. [35] and Strömberg et al. [37] is focused on vehicle automation, the greatest technological challenge for road transport in the next decades. Given the pace of technology development, fully-automated vehicles will inevitably arrive on public roads in a few years from now [19]. However, as full automation will not become common and affordable overnight, for the time being, numerous efforts are being made to evaluate the safety of partially- and conditionally-automated systems. Soares et al. [35] presented a literature review of these recent efforts, focused on the analysis of human capabilities to recover the manual control of an automated vehicle (AV) when the system fails or when it is unable to cope with certain (more or less critical) traffic situations. Considering that the takeover manoeuvre is arguably the most relevant new risk introduced by vehicle automation, and that testing it on real roads poses significant ethical and practical challenges, most research has been conducted in driving simulators, testing situations with varying levels of complexity. It was found that the probability of serious incidents and accidents increases with the complexity of the situation, but also with the degree to which the driver is allowed to exempt himself/herself from monitoring the driving task and engage in other activities at the wheel.

Strömberg et al. [37] developed a technology acceptance study using focus groups that confirmed these safety concerns and other fears reported in the literature, such as possible automation malfunctions, hacking, data leaks, lack of legislation, misuse, and liability if anything goes wrong [7, 10, 30, 33]. The focus groups, consisting of three groups of stakeholders (future users, urban planners, and technology developers), further discussed the implications of a large-scale deployment of fully-automated vehicles at an urban scale. All groups agreed that technology will not be affordable from the beginning, leading once again to the provision of an elitist transport mode [1]. However, they also agreed that the future dissemination of AVs (and the investments needed to make it happen) could conflict with the objectives of reducing car reliance, emissions, and congestion. Users were also concerned that investments in AVs could be prioritized over investments in more sustainable alternatives, such as the railway and the cycling networks.

## Conclusions

Private cars remain attractive for a large share of the population who can afford their costs, mainly due to comfort and flexibility benefits and/or poor access to alternative modes. In fact, car trips represent more than 80% of all land transport (measured in passenger-km) in the EU. This value has remained stable over the last 20 years, despite the proliferation of new transport options and digital services with potential to avoid many trips.

The COVID-19 pandemic brought a new reality that increased the gap between the high and the low ends of social stratification, also in relation to the transport mode choice. In fact, it has been observed that the reduction of car trips has been entirely recovered in only 1 year, whereas public transport trips have not. Passenger volumes by car (as well as the related undesired externalities, such as pollutant emissions and road fatalities) have started to raise again, and, as the pandemic is still evolving, the long-term impacts on the transport sector are uncertain.

Accessibility gaps are ubiquitous at all scales. The availability of public transport infrastructure to cope with the increasing complexity of people’s daily activities is still deficient, especially in rural and suburban areas. Emerging on-demand riding services and the availability of shared vehicles, such as cars, bikes, and micro-mobility ones, have a potential to bridge up the gap with car-based services, provided that these would be kept affordable for all, and potentially capturing users from more sustainable modes (e.g., to feed public transport stations and terminals).

Emerging big data storage and processing tools have been consolidating an integrated analysis of different transport modes, with a potential to improve tactical and strategic transport planning actions oriented to shift behaviours toward more sustainable choices. Younger generations may be supportive of new technologies but are not necessarily giving up on driving. There are still major safety concerns towards AVs regarding their interactions with the human user and their capabilities to deal with complex traffic situations, especially for intermediate levels of automation. Once the safety problems are solved, the integration of AVs in the transport ecosystem will be quite challenging over fears of increased congestion, pollution and competition with more sustainable modes. The potentially high costs of technology and the difficulties of users in understanding/using it also raise concerns of social exclusion.
